# Australian Clinicians’ Capabilities, Opportunities, and Motivations in Implementing Exposure and Response Prevention for Youth with Obsessive-Compulsive Disorder: An Exploratory Study

**DOI:** 10.3390/children12020156

**Published:** 2025-01-28

**Authors:** Jason I. Racz, Iain E. Perkes, Andrea Bialocerkowski, Katelyn M. Dyason, Jessica R. Grisham, Matthew L. McKenzie, Lara J. Farrell

**Affiliations:** 1 School of Applied Psychology, Griffith University, Southport, QLD 4222, Australia; 2 Discipline of Paediatrics & Child Health, School of Clinical Medicine, Faculty of Medicine & Health, University of New South Wales, Sydney, NSW 2052, Australia; 3 Discipline of Psychiatry & Mental Health, School of Clinical Medicine, Faculty of Medicine & Health, University of New South Wales, Sydney, NSW 2052, Australia; 4 Department of Psychological Medicine, The Sydney Children’s Hospitals Network, Westmead, NSW 2145, Australia; 5 Griffith Health, Griffith University, Southport, QLD 4222, Australia; 6 School of Psychology, University of New South Wales, Sydney, NSW 2052, Australia; 7 Centre for Mental Health, Griffith University, Southport, QLD 4222, Australia

**Keywords:** implementation, cognitive-behavioral therapy, exposure and response prevention, pediatric, obsessive-compulsive disorder

## Abstract

**Objectives:** Informed by implementation science, this exploratory study examined the capabilities, opportunities, and motivations of Australian mental health clinicians (*N* = 38) associated with the implementation of exposure and response prevention (ERP) for youth (i.e., children and adolescents) with obsessive-compulsive disorder (OCD). It also explored how the capabilities and motivations of mental health clinicians untrained in ERP for youth (i.e., typical clinicians; *n* = 25) differed from clinicians experienced in the use of ERP for youth (i.e., experienced clinicians; *n* = 13). **Methods:** Questionnaires were administered to the entire sample alongside observational role-plays, which assessed observed adherence and competence delivering ERP against published best-practice standards among available participants. **Results:** In the whole sample, the reported time dedicated to implementing ERP was associated with a range of factors relating to capabilities, opportunities, and motivations to implement ERP. Experienced clinicians had greater knowledge, adherence, competence, and self-reported confidence using ERP and fewer negative beliefs about ERP relative to typical clinicians. They also intended to dedicate greater time to implementing within-session ERP for youth and reported greater within- and between-session time spent doing so compared to typical clinicians. The time intended to dedicate to implementing between-sessions ERP did not significantly differ between the clinician groups. **Conclusions:** In summary, compared to typical clinicians, experienced clinicians appeared to possess greater levels of capabilities and motivations to implement ERP for youth with OCD. Future research examining barriers and facilitators of ERP implementation in community settings and identifying effective strategies to improve it would benefit service provision and, ultimately, outcomes for youth.

## 1. Introduction

Obsessive-compulsive disorder (OCD), with its distressing and intrusive thoughts and repetitive, time-consuming rituals or behaviours [1], has significant and varied consequences for the one-in-thirty youth (i.e., children and adolescents) who experience it in any 12-month period [2]. OCD in childhood and adolescence is associated with a myriad of deleterious consequences, including impairments and disruptions for youth and their parents at school, work, and home [3,4]. Moreover, it is associated with the presence of additional psychiatric diagnoses [3]. Although meta-analyses have demonstrated the course for OCD in childhood and adolescence need not be chronic, it frequently persists at a clinical level in more than 40% of youth and at a subclinical level in 60% [5], with most cases demonstrating a chronic, fluctuating course [6]. Importantly, younger age and baseline anxiety and obsessive-compulsive symptoms in youth all predict greater obsessive-compulsive symptom severity over time [7], while lower symptom severity predicts better treatment response [8], highlighting the need for the implementation of early, effective treatment. Despite this, recent estimates suggest that most patients go undiagnosed for over a decade following onset [9] and that hospitalisation rates and length-of-stay in youth approximate those of psychotic disorders, despite high levels of treatment compliance and engagement [10]. The treatment gap in the provision of evidence-based care for youth with OCD remains underexplored in the Australian context.

### 1.1. Exposure and Response Prevention for Youth with OCD

Practice parameters recommended cognitive-behavioural therapy (CBT) with emphasis on exposure and response prevention (ERP) as the first-line monotherapy for mild to moderate OCD among children and adolescents and in combination with medication, namely serotonin reuptake inhibitors, for moderate to severe presentations [11]. These guidelines are supported by a robust evidence base of controlled trials. For example, a meta-analysis by Watson and Rees [12] of five randomised controlled trials found a large pooled effect of CBT on OCD symptoms across 161 youth aged 19 years and under. This effect exceeded that of pharmacotherapy. Another meta-analysis of 10 randomised controlled trials of CBT found remission of obsessive-compulsive symptoms in 57% of youth aged 17 years or under with OCD [13]. These treatment outcomes also demonstrated robustness in the presence of commonly comorbid presentations. For example, even greater effect sizes have been observed in the presence of comorbid anxiety and tic disorders, likely due to higher levels of the shared underlying fear-based psychopathology encouraging a more robust treatment response [13].

ERP directly targets the underlying fear via gradually and repeatedly confronting feared situations that trigger obsessions, whilst simultaneously resisting the compulsions that characterise OCD [14] to inhibit fear-based psychopathological learning and encourage the primacy of new safety-based learning [15]. Meta-analyses in youth with OCD have found CBT trials emphasising ERP boast larger treatment effects relative to those emphasising cognitive therapy; however, these differences have been non-significant, likely due to the small number of trials emphasising cognitive therapy [13]. However, longitudinal analyses in anxious youth have indicated that the implementation of exposure therapy is related to significant symptom improvement after controlling for the contribution of cognitive therapy [16] and that it is more efficient without the use of anxiety management strategies [17]. As such, ERP is widely recognised in treatment guidelines as the core component of the gold-standard treatment of OCD in youth using CBT [11,18].

### 1.2. The Underutilisation of ERP for Youth with OCD

Despite the decades of scientific research supporting the implementation of ERP for OCD [13], it is acknowledged that there is an issue with its large-scale dissemination and utilisation in community mental health settings [19]. A study of 107 multidisciplinary mental health clinicians working across private and public health services in the United Kingdom found that less than half reported always using therapist-assisted in vivo exposure for the treatment of youth with OCD [20]. Similar findings have emerged in the United States, where therapists spend less than a third of treatment time implementing within-session exposure for children and adolescents with OCD and spend significantly more time implementing suboptimal exposure as client self-directed tasks [21]. While the literature examining the implementation of ERP for youth specifically is relatively sparse, the wider literature suggests ERP for OCD is underutilised across the lifespan at a concerning rate [20,21,22], is implemented more frequently as between-session homework than as within-session tasks [20,21,22,23], or is implemented with suboptimal fidelity, for example by allowing safety behaviours [24,25].

These international findings consistently indicate a research-practice gap, and preliminary Australian research has suggested a similar issue. Australian data collected from a small pilot sample of mental health clinicians receiving training in CBT-ERP [26] suggested that among clinicians (*n* = 11) who had treated a young person with OCD in the previous 12 months, approximately three out of four implemented ERP but spent only 17% of within-session and 34% of between-session treatment time doing so. For the treatment of adults with OCD in Australia, another study found that although 63% of psychologists utilise therapist-assisted ERP at least half of the time, only one in five of them consistently implement ERP within-sessions [27].

Other indicators have corroborated the existence of a treatment gap in the community. An analysis of admissions at two paediatric inpatient units over a six-year period in Australia’s largest provider of children’s health services found that OCD was associated with a disproportionately high voluntary admission rate, length of stay, and readmission rate [10]. These findings suggest that young Australians with OCD are experiencing significant difficulty accessing effective evidence-based treatment in their community, including optimally delivered ERP. Despite this, no research has explored the factors influencing the implementation of ERP for youth in the Australian context. The need for this research is significant due to important differences in the Australian implementation context, compared to other international jurisdictions, that may be influential, such as the emphasis on CBT during clinical training [28].

### 1.3. Conceptualising the Implementation of ERP for Youth with OCD in Australia

The dissemination and implementation of evidence-based practices in mental healthcare is a widespread and longstanding issue [29]; although, in recent decades, considerable efforts have been directed at addressing this [30]. This includes efforts aimed at understanding and improving the implementation of exposure therapy for anxiety disorders [23,31]. However, the disproportionate focus of psychological research on clinical treatment outcomes, at the expense of evaluating the implementation of these treatments in routine community practice, remains a significant issue in clinical psychology [32]. Indeed, a paucity of research has sought to understand the factors driving the underutilisation of ERP for youth with OCD internationally and in Australia [33]. Moreover, the literature has rarely capitalised on evidence-based methods of conceptualising the implementation of ERP [33], an issue in the psychotherapy literature broadly [30]. This results in narrow or poorly defined interventions for implementation that produce a poor return-on-investment [34].

Using implementation science, or the use of the scientific method to promote the uptake of evidence-based practice in routine practice [35], provides an approach to understanding and addressing such research-practice gaps efficiently. One implementation framework, the COM-B system, suggests that capabilities, opportunities, and motivations influence and are, in turn, influenced by clinicians’ implementation behaviour [36]. The existing literature examining ERP implementation has suggested that clinicians’ capabilities and motivations represent salient [33] and amenable contributors to the underuse of ERP [26] and exposure therapy more broadly [37,38]. While opportunity lies outside of the individual clinician [36] and therefore requires a different approach to intervention, understanding the influence of factors relating to the clinician’s practice context and support, as well as the client, is critical to guiding the development of such interventions [39]. Overall, seeking to improve the understanding of the factors related to the implementation of ERP for paediatric OCD in the Australian mental health context is required to improve access to effective evidence-based care delivered in community settings to reduce unnecessary and costly hospital admissions.

One approach to understanding barriers to implementation is to identify the capability and motivational factors that may be driving the underuse of ERP in routine community practice. In recent years, the literature has highlighted that ERP for OCD is a specialised treatment requiring specialised training for optimal implementation [40]—this may be one potential barrier to the utilisation of ERP by clinicians. Drawing comparisons between typical clinicians (i.e., those without specialised ERP training) working in community settings relative to experienced clinicians with specialised training in the delivery of ERP for youth with OCD may provide insight into this area while generating hypotheses around which factors represent targets for intervention in community clinicians.

Two important factors relating to capabilities [36] that few studies in the international exposure therapy implementation literature have examined are adherence and competence to practice standards [38]. This is despite the importance of fidelity as an indicator of adequate dissemination [32]. Recent efforts by an international team of experts in CBT for youth with OCD (the International Obsessive-Compulsive Disorders Accreditation Task Force) have identified core knowledge and competency standards that should be met by providers who self-identify as specialists in the assessment and treatment of youth with OCD [19]. Exploring adherence and competence to these standards represents a novel and crucial metric to determine Australian community clinicians’ capabilities to implement ERP.

### 1.4. The Current Study

This study builds on previous investigations into factors contributing to the underutilisation of ERP in Australia through the lens of implementation science. This exploratory study aimed to (a) conduct an initial exploration of the factors associated with Australian clinicians’ implementation of ERP for youth (i.e., children and adolescents) across the COM-B system domains [36]. Specifically, factors were examined that related to clinician demographics (i.e., age; gender), *capability* (i.e., education; years of experience as a clinician; experience treating youth with OCD; training in specialised CBT for OCD; knowledge of ERP for youth; observed adherence and competence in delivering ERP for youth), *opportunity* (i.e., working in private practice or other settings; working with adolescents or children; recent supervision in the treatment of youth with OCD), and *motivation* (i.e., profession as a psychologist or other; negative beliefs about and confidence using ERP for youth; time intended to dedicate to implementing within- and between-session ERP for youth). Additionally, participants self-reported their ERP *implementation behaviour* (i.e., the reported time dedicated to implementing within- and between-session ERP for youth). Utilising a between-subjects design, this study then aimed to (b) explore how factors related to Australian typical mental health clinicians’ *capabilities*, *motivations,* and *implementation behaviour* differ relative to clinicians who are experienced in ERP implementation for youth (i.e., possessing specialised training and practice in ERP for OCD). It was broadly hypothesised that clinicians possessing greater capability, opportunity, and motivation to implement ERP would be associated with greater reported implementation behaviour. Moreover, it was expected that the typical clinician would possess lower capability and motivation to implement ERP, as well as lower reported implementation behaviour, relative to experienced clinicians.

## 2. Method

### 2.1. Participants

Participants (*N* = 38) consisted of Australian mental health clinicians who were registered with the appropriate industry body for their profession and were working with child and/or adolescent clients. There were two subsamples: (a) typical clinicians (*n* = 25) recruited via convenience sampling; and (b) experienced clinicians possessing specialised training and practice in ERP for youth with OCD (*n* = 13) recruited via purposive sampling. Typical clinicians confirmed they had no additional training in ERP for youth with OCD beyond their graduate clinical training programs and brief professional development workshops; they also reported that they had not received more than 10 hours of supervision in the last 12 months for the treatment of youth with OCD. In contrast, status as an experienced clinician was operationalised as having received specialised training in CBT-ERP via a graduate-level clinical placement in a university specialist OCD treatment program delivering CBT-ERP, a postdoctoral position with a focus on CBT-ERP for OCD, and/or working at a private specialist clinic focusing on the treatment of youth with OCD using CBT-ERP.

#### 2.1.1. Typical Clinicians

The typical clinicians (80% female) worked in mental health services in the community (60%), private practice (28%), hospital (8%), or other settings (4%). They were either psychologists (80%) or social workers (20%), and most (76%) worked predominantly with adolescents (i.e., 12 to 18 years old) relative to children (i.e., <12 years old). They averaged 36 years old (*SD* = 12) with 9 years of experience (*SD* = 11) as a clinician and possessed either a master’s (72%) or a bachelor’s degree (28%). Nearly all (92%) had no previous training in specialised CBT for OCD beyond that provided in their postgraduate training, and the remainder reported only receiving brief training via a workshop. Most (56%) had not received any recent supervision for the treatment of youth with OCD, and those who had reported receiving, on average, 5 hours in total (*SD* = 3). Inexperience was confirmed by the subsample’s self-reported experience treating youth for OCD as *limited* (*M* = 2.36, *SD* = 1.19), and none rated themselves as *very experienced/expert.*

#### 2.1.2. Experienced Clinicians

The experienced clinicians (85% female) worked in mental health services in the community (23%), private practice (31%), hospital (15%), or other settings (31%). They were all psychologists, and most (77%) worked predominantly with adolescents compared to children. They averaged 35 years old (*SD* = 9) with 10 years of experience (*SD* = 8) as a mental health clinician and possessed either a doctoral (39%), master’s (46%), or bachelor’s degree (15%). The verification of experience was corroborated by the subsample’s self-reported experience treating youth for OCD as *experienced but not expert* (*M* = 3.92, *SD* = 0.64), and all reporting at least *some experience.*

### 2.2. Procedure

#### 2.2.1. Ethical Approval

The study procedures were approved by the human research ethics committees of the Sydney Children’s Hospitals Network (2022/ETH01213) and Griffith University (2022/756 and 2023/633). All participants consented to the study procedures.

#### 2.2.2. Recruitment

Typical clinicians were recruited via convenience sampling prior to completing a training workshop in CBT-ERP for youth as part of a previous study evaluating training outcomes [26] or by responding to recruitment efforts targeting relevant workplaces and online communities. Experienced clinicians were recruited using purposive sampling, which involved the research team independently approaching clinicians identified as possessing significant experience in delivering ERP for youth. Two experienced clinicians were identified from the typical clinician sample as possessing significant experience prior to engagement in the study.

#### 2.2.3. Data Collection

Data were collected via an online questionnaire using REDCap electronic data capture tools [41], and a subset of available typical (*n* = 14, 56%) and experienced clinicians (*n* = 12, 92%) completed role-plays with professional actors. Microsoft Teams was used to conduct and audio-visually record the role-plays; further information is provided below. 

##### Role-Plays Assessing Adherence and Competence Delivering ERP for Youth

Participants’ observed adherence and competence delivering ERP for youth per published practice standards for CBT-ERP for paediatric OCD [19] were assessed utilising standardised role-plays. The research team developed three standardised 20-minute cases and accompanying scripts to depict clinical OCD presentations typically observed in this population. Client details (e.g., 12-year-old client, living situation) and the therapeutic context (i.e., a follow-up telehealth appointment) were kept consistent across cases. 

Participants were randomly assigned to one of the three cases based on their availability to attend a role-play. Briefing documents, which outlined the context and the client’s background and presentation (i.e., obsessions and compulsions) were provided for participants to review for up to five-minutes before the role-play. Participants were instructed to assume they had already established rapport with the client, completed an assessment, and provided psychoeducation. Their task involved: (a) summarising psychoeducation and the rationale for ERP; (b) collaboratively designing an ERP task with the client; and (c) conducting an ERP task with the client. The development and content of these cases was detailed in Racz et al. [26] where the cases and briefings were evaluated by the research team for consistency and piloted with graduate clinical psychology students and paid actors to ensure the clarity and consistent application of the instructions.

The role-plays were conducted using experienced professional actors who portrayed the young client using the semi-structured scripts. The actors were employed from a university human patient simulation program, where they had received relevant training for clinical training role-plays within university health programs. The actors were provided with similar briefing documents that provided the information detailed above, alongside additional information on paediatric OCD and its treatment, consistent with psychoeducation typically delivered in early treatment sessions [14]. The actors’ briefing documents also outlined the character’s treatment goal, potential exposure steps, and expected behavioural responses to completing tasks (e.g., distress levels, anxious behaviour, reassurance seeking, distraction attempts). Before each set of role-plays, the actors were provided a 30-minute briefing on the cases and scripts conducted by an expert in OCD in youth (JIR) to enhance authenticity and ensure consistency in their performance. 

### 2.3. Measures

#### 2.3.1. Self-Report Measures in Online Questionnaire

##### Clinician Demographics

Ten items assessed both personal and professional demographic information. Age and clinical experience were measured in years. Gender was measured as male, female, non-binary, or prefer not to say. Practice setting (e.g., community, school, hospital), profession (e.g., psychologist, psychiatrist, mental health nurse), and typical client age group (i.e., children or adolescents) were answered by selecting the most appropriate category. Education was rated on a 7-point scale (1 = *Year 10 or below* to 7 = *doctoral degree*). Experience treating youth with OCD was rated on a 5-point Likert scale (1 = *very inexperienced* to 5 = *very experienced*). Two items were quantified using a dichotomous scale (*yes*, *no*) and assessed (a) having completed at least one day of specialised CBT training for OCD (e.g., postgraduate training, conference attendance, online workshop) and (b) having received supervision in the 12 months prior, specific to the treatment of youth with OCD.

##### Knowledge of ERP for Youth

A 20-item multiple-choice quiz with four response choices assessed participants’ knowledge of ERP for youth in clinical practice. Consistent with previous evaluations of skills in exposure therapy [38], this scale was developed for a previous study, where it demonstrated sensitivity to training [26]. The items developed possessed acceptable internal consistency (α = 0.70). Correct responses were summed to produce a knowledge of ERP for youth score (range of 0 to 20).

##### Negative Beliefs About ERP for Youth

The 21-item Therapist Beliefs about Exposure Scale (TBES; [42]) was used to measure therapists’ negative beliefs about ERP. Minor wording changes were made to reflect the study’s focus on ERP for youth, not exposure therapy broadly. The items (e.g., “most children and adolescents have difficulty tolerating the distress exposure and response prevention evokes”) were measured on a 5-point Likert scale (1 = *disagree strongly* to 5 = *agree strongly*). Item scores were averaged to produce a negative beliefs about ERP for youth score (range of 21 to 105). This scale demonstrated excellent internal consistency in the current sample (α = 0.90).

##### Confidence Using ERP for Youth

Two items assessed participants’ confidence in their ability to assist and support youth during ERP. The items were adapted from a parent exposure self-efficacy scale [43] to assess participants’ confidence using ERP for youth. Items were re-worded to measure confidence helping youth manage their OCD during treatment (i.e., “how sure are you that you could assist and support child and adolescent clients in their exposure steps in fighting OCD” and “how sure are you that you could handle your own discomfort/concerns in supporting child and adolescent clients in their exposure steps in fighting OCD”) on a 9-point Likert scale (0 = *not at all sure* to 8 = *extremely sure—I know I could*). The items were summed to produce a confidence using ERP for youth score. The scale demonstrated good internal consistency (α = 0.86).

##### Time Intended and Dedicated to Implementing ERP for Youth

Two items developed for a previous study [26] measured the proportion of time (0–100%) participants intended to dedicate to implementing ERP for youth with OCD as (a) *therapist-assisted within-session tasks* (i.e., “going forward, what proportion of treatment with children and adolescents with OCD do you intend to spend delivering within session therapist-assisted ERP tasks?”) and (b) *therapist-instructed between-session homework* (i.e., “going forward, what proportion of the homework you assign to children and adolescents with OCD do you intend to spend on therapist-instructed ERP tasks?”).

One item dichotomously developed for the study asked (*yes*, *no*) whether participants had recently treated any youth for OCD (i.e., “in the past 12-months, have you treated any children and/or adolescents for obsessive-compulsive disorder?”). If answered *yes*, participants were requested to provide the self-reported proportion of time (0–100%) dedicated to implementing ERP for youth with OCD as (a) *therapist-assisted within-session tasks* (i.e., “in the past 12-months, what proportion of your treatment with children and adolescents with OCD has been spent delivering within session therapist-assisted ERP tasks?”) and (b) *therapist-instructed between-session homework* (i.e., “in the past 12-months, what proportion of the homework you have assigned to children and adolescents with OCD has been spent on therapist-instructed ERP tasks?”).

##### Attention Checks

Four checks were included throughout the questionnaire at each time point to assess attention to scale instructions and items. Based on established techniques [44], these checks assessed whether participants had attended to instruction (two items; e.g., “please answer A for the first item”) and item wording (two items; e.g., “I was born on February 30th”). These checks were accompanied by explanatory text (e.g., “to show that you have read this instruction”). The inclusion of these checks was expected to result in overall improvements in data quality [44].

#### 2.3.2. Role-Play Measures

##### Adherence and Competence Delivering ERP for Youth

The Adherence and Competence with ERP for Youth Scale (ACEYS), coded against current practice standards for specialised CBT for youth with OCD [19], was employed to assess participants’ observed adherence and competence delivering ERP for youth during the standardised role-plays. The development of the ACEYS was detailed by Racz et al. [26]. Practice standards deemed essential to ERP and relevant to the tasks participants were requested to complete in the role-play were measured across 14-items (e.g., “provided assistance and modelling as a therapist”). Each item was scored on two scales: (a) observed adherence (0 = *absent*, 1 = *present*) and (b) observed competence delivering ERP for youth (1 = *poor*, 2 = *satisfactory*, 3 = *good*) for only those adherence items that were present. 

The role-plays were scored on the ACEYS minute-by-minute from the original audio-visual recording as recommended by Waltz et al. [45]. Over subsequent minutes observed competence for any skills previously scored was updated to reflect any additional demonstration. This approach meant that observed adherence was scored as either absent or present at any point during the role-play, and observed competence was a global score summarising all demonstrations of the skill throughout. For analyses, adherence scores were summed to provide a total adherence score, and competence scores were averaged to provide an overall competency score.

The role-plays were scored by the primary author (JIR), while another author (MLM), who was blinded to the subsample, independently scored 25% of the role-plays. Both possessed clinical experience working with youth with OCD. Any discrepancies were discussed and consensus was reached. Using a single rater, consistency, two-way random-effects model, the ACEYS demonstrated moderate to good interrater reliability [46] across observed adherence (ICC = 0.70) and competence (ICC = 0.80; after controlling for variance in adherence) subscales. 

##### Role-Play Fidelity

To ensure consistency, role-play fidelity (i.e., the extent to which the actor’s performance approximated reality [47]) was assessed using nine items (e.g., “the client appeared authentic”) measured on a five-point Likert scale (1 = *completely disagree*; 5 = *completely agree*). Four items were reverse scored, and all items were averaged to produce a role-play fidelity score. These items were adapted from a scale used to assess simulation fidelity in other allied health settings [48]. This measure was completed by both the participant via a standalone online questionnaire after completing the role-play and by an observer at the end of scoring each role-play recording, per the procedure used for the ACEYS. The participant-rated scale demonstrated acceptable internal consistency (α = 0.78), while the observer-rated scale possessed moderate interrater reliability (ICC = 0.69) in a single rater, consistency, two-way random-effects model [46].

## 3. Results

### 3.1. Overview of Analyses

Before conducting analyses, the data were cleaned, and statistical assumptions were assessed. Missing data were minimal (<1% at item-level; 10.41% at construct-level) and found to be missing completely at random, χ^2^(37) = 30.21, *p* = 0.778. Missing item-level data were replaced by the participants’ scale mean, while the remaining construct-level data were excluded pairwise due to the small sample size [49]. Extreme scores on constructs were checked, two of which (0.24% of construct-level data) were removed to reduce their influence, as the participants’ response patterns were observed to be inconsistent. Analysis of the attention checks indicated that, as is often the case with questionnaires [44], most participants (i.e., 63%) did not attend to scale instructions. However, all participants consistently attended to item instructions, so no corrective action was taken. Non-parametric Kruskal-Wallis ANOVAs confirmed there were no significant differences (all *p*s > 0.05) across relevant outcome measures (i.e., observed adherence and competence delivering ERP for youth) or on participant-rated and observer-rated role-play fidelity between the role-play case examples and actors. Given poor normality and the small sample sizes, all analyses were conducted using non-parametric tests.

A series of Kendall’s Tau-b correlation analyses were conducted to examine the association between relevant *capability*, *opportunity*, and *motivation* variables and *implementation behaviour* (i.e., the self-reported time dedicated to implementing ERP for youth) in the sample. Then a series of Mann-Whitney *U* Tests were used to assess differences on relevant *capability* and *motivation* variables between the subsamples of typical and experienced clinicians. The shape of the distributions differed, as was expected from the subsamples, and therefore mean ranks rather than medians were compared [50]. All other assumptions for the planned analyses were met.

### 3.2. Associations with Time Dedicated to Implementing ERP for Youth

Table 1 presents the Kendall’s Tau-b association between the reported time dedicated to implementing ERP for youth and other relevant variables in the whole sample (*N* = 38). It demonstrates that greater reported time dedicated to implementing ERP for youth with OCD both within-session (*Mdn* = 40, *IQR* = 68) or between-session (*Mdn* = 38, *IQR* = 78) was significantly associated with a range of variables relating to clinicians’ *capabilities* (i.e., training in specialised CBT for OCD, greater observed adherence and competence delivering ERP), *opportunities* (i.e., recent supervision in the treatment of youth with OCD), and *motivations* to implement ERP (i.e., less negative beliefs about ERP, greater confidence using ERP, greater time intended to dedicate to implementing within-session ERP). Some variables were only related to greater within-session ERP implementation, including fewer years of experience as a clinician, greater knowledge of ERP for youth, a profession as a psychologist, and level of education. Conversely, some variables were only associated with greater between-session ERP implementation, including working with adolescents (relative to children), younger clinician age, and greater time intended to be dedicated to implementing between-session ERP for youth.

### 3.3. Differences Between Typical and Experienced Clinicians

A series of Mann–Whitney *U* tests were conducted to compare the typical (*n* = 25) and experienced subsamples (*n* = 13) across key variables related to *capabilities*, *motivations*, and *implementation behaviour*, as presented in Table 2. These analyses indicated that, relative to typical clinicians, experienced clinicians ranked higher in knowledge of ERP, observed adherence and competence delivering ERP, confidence using ERP, the time intended to dedicate to implementing within-session ERP for youth, and both the reported time dedicated to actually implementing within- and between-session ERP. They also ranked lower in negative beliefs about ERP for youth, relative to typical clinicians. While the mean ranks, not medians, were compared in the analysis [50], relevant medians and interquartile ranges are presented for reference in Appendix A.

## 4. Discussion

This study aimed to explore the correlates of clinicians’ implementation of ERP for youth with OCD, informed by implementation science (i.e., the COM-B system [36]). Findings indicated that a range of factors related to clinicians’ *capabilities*, *opportunities*, and *motivations* to implement ERP for youth are significantly associated with their *implementation behaviour* (i.e., the time dedicated to implementing it both within- and between-sessions). These results provide preliminary evidence suggesting that all three factors of the COM-B system are associated with the underutilisation of ERP for youth in Australia and are potential targets for cost-effective implementation interventions. Although previous studies have piloted improving capabilities and motivations via training [26], the current findings emphasise the need to also improve the opportunities clinicians have to implement ERP, including relevant clinical supervision. This study also found that predominantly working with adolescents, rather than children, was moderately associated with greater between-session time spent implementing ERP but not within-session. This finding could reflect competing contextual factors (e.g., treatment priorities) that reflect implementation opportunities or that clinicians may be less likely to provide exposure therapy to children than to adolescents for reasons such as their perceived lack of resiliency [51].

In the current study, the factors related to opportunities within the COM-B system were limited, but these results indicate that further research exploring a broader range of factors in this domain is important. Other implementation science frameworks that map onto the COM-B system may help identify theoretically relevant determinants warranting exploration. For example, the Theoretical Domains Framework [52], identifies that both social influences (i.e., interpersonal processes causing individuals to change their thoughts, feelings, or behaviour) and environmental context and resources (i.e., situational or environmental circumstances directly or indirectly related to behaviour) are domains related to opportunity. Factors under these domains, including formal and informal supervision (e.g., Keleher et al. [20]), social pressure within organisations to adopt treatment approaches, and the resources (e.g., time, stimuli) accessible to clinicians within their work environment (e.g., Becker-Haimes et al. [53]), have been examined elsewhere in the exposure therapy implementation literature with mixed results [33] and would represent potential determinants to consider in the implementation of ERP for Australian youth. Considering the range of potential barriers to utilisation, qualitative or mixed-method research may also provide important insight [54] into identifying the relevant factors influencing clinicians’ opportunities to implement ERP for youth.

To the authors’ knowledge, interviews have not been used to explore, in depth, the perceptions of clinicians toward their opportunities to implement ERP for youth. However, adjacent quantitative studies have highlighted the need for explorative qualitative research in this area. For example, Keleher et al. [20] found that among 107 clinicians in the United Kingdom, factors such as session length and disapproval from parents or colleagues were barriers to implementation. Similar methods have also identified that over one-fifth of clinicians reported support and access to stimuli for exposure tasks as necessary for ERP implementation across the lifespan [55]. For the treatment of anxious children, a qualitative analysis of interviews conducted with 50 therapists in the United States identified various organisational factors, such as working in a school and a lack of internal support, as barriers to CBT implementation [56]. Qualitatively exploring such factors prior to quantitatively prioritising them as intervention targets would enable more efficient resource expenditure when designing interventions to address this research-practice gap.

The current study also aimed to explore if factors relating to *capability* and *motivation* to implement ERP for youth and *implementation behaviour* differed between Australian typical and experienced clinicians. The findings suggested that experienced clinicians possess significantly higher capabilities and motivations across a wide range of variables and, in turn, implementation behaviour. To the authors’ knowledge, these findings are the first in the literature to demonstrate that the capabilities and motivations to implement ERP, alongside utilisation, in clinicians with specialisation in ERP for youth nearly ubiquitously surpass those possessed by typical clinicians in the Australian context. This study, therefore, provides evidence that experienced clinicians with specialised training, whom young Australians with OCD are unlikely to first access for treatment, are better equipped on a range of factors associated with the implementation of ERP, the empirically supported first-line treatment.

There were also significant group differences in adherence and competence on practice standards, assessed using observational data. While typical clinicians adhered to most practice standards relevant to the delivery of ERP for youth, the median adherence for experienced clinicians suggested near-perfect fidelity to treatment standards (see Appendix A). This mirrors similar findings in the anxiety disorders literature. McLeod and colleagues [57] found that when treating youth with anxiety, community therapists demonstrated significantly lower adherence and competence across treatment components relative to benchmarks established by therapists in research settings. However, when delivering exposure treatment components, a larger deficit in adherence was found for community therapists. The current study suggests a similar issue is likely impacting the treatment of youth with OCD by typical clinicians using ERP, even when compared to experienced clinicians also predominantly engaged in community-based practice.

The finding that competence in ERP delivery was significantly higher for experienced clinicians suggests that, even when typical clinicians implement ERP in line with practice standards, experienced clinicians still do so at a higher level of competence. Importantly, however, it is not known if differences in clinician competence translate to significantly different treatment outcomes for youth. This gap in the existing literature warrants exploration using observational data collected during psychotherapy sessions delivered to young clients with OCD. Although studies have shown that the treatment protocol for ERP, not the therapist, is the best predictor of treatment outcomes when using ERP [58], the current findings suggest it is likely that in routine practice, where protocol fidelity is not monitored, typical clinicians may be less likely to demonstrate fidelity. While this can likely be improved by disseminating implementation interventions such as clinician training [26], such interventions cannot reach all clinicians, and therefore, the findings support the movement in the international community toward establishing criteria and processes for certifying mental health clinicians who possess the capabilities associated with delivering CBT-ERP for youth with fidelity [19]. Overall, these findings suggest that the implementation of ERP for youth benefits from specialist capabilities above and beyond those acquired during the normal clinical training possessed by typical clinicians in Australia. This has implications for youth with OCD, especially those with moderate to severe symptomatology, who possess high rates of inpatient admissions [10], likely, in part, due to difficulty accessing effective care in the community [59].

### 4.1. Limitations and Future Research

Although the current study represents the first exploration of how capabilities, opportunities, and motivations are associated with the implementation of ERP for youth among Australian clinicians and how these differ between typical and experienced clinicians, there are several limitations. Foremost, while the study used implementation theory to ensure theoretical coverage of implementation determinants and to guide the interpretation of analyses, the domains of capability and motivation within the COM-B system [36] were not exhaustively examined. The absence of qualitative data prevented the open exploration of additional implementation determinants that may have enriched the quantitative results obtained. Moreover, variables relating to the domain of opportunity tended also to be related to implementation, and as such, future research should emphasise other explorative methods, including qualitative or mixed-method approaches, to identify the most relevant targets for intervention in this area.

Additionally, a high proportion of typical clinicians drawn were from public mental health services, where competing demands (e.g., comorbidities) may hinder opportunities to implement ERP for youth with OCD. It is possible that such contextual barriers to implementing ERP may also hamper capability and motivation by reducing clinicians’ opportunities to improve their skills and beliefs around ERP through experience utilising it. Research should also consider examining the pathways through which implementation determinants (i.e., capability, opportunity, and motivation [36]) influence each other and, in turn, ERP implementation.

Beyond this, the current study was adequately powered to detect medium effects and to provide a novel hypothesis-generating framework for exploration in future studies. However, the small sample size, especially for experienced clinicians who were difficult to identify for recruitment, meant that smaller effects may have gone undetected and the findings’ generalisability may be limited. As such, replication in larger representative samples is necessary. Finally, the method was strengthened by the novel examination of the capabilities of clinicians in structured role-plays, using observed adherence and competence against international practice standards [19], which found significant differences between experienced and typical clinicians. This method, however, was unable to determine the threshold for adherence and competence that are necessary for effective treatment and, as a result, if the differences observed translate to variable treatment outcomes for youth with OCD. Benchmarking these implementation outcomes in routine practice is an important goal for clinical psychology research broadly [32].

### 4.2. Implications and Conclusion

As the first study to examine how implementation determinants related to the implementation of ERP for youth with OCD in Australia and how they differ between typical and experienced clinicians, the results of this study support that clinicians’ capabilities, opportunities, and motivations play significant roles in ERP implementation. Capabilities and motivations benefited from experience beyond that possessed by the typical community clinician. It is likely these factors are contributing to the intention-practice gap in the use of ERP for youth observed in Australian mental health clinicians [26]. They are also likely limiting the access young Australians with OCD have to effective treatment for their symptoms. This exploratory study provides support for the mounting evidence that specialist providers best treat OCD in line with guidelines. Future studies should focus on benchmarking the levels of capabilities and motivations necessary to maximise treatment outcomes for youth receiving ERP for OCD, while also identifying and addressing the barriers that influence clinicians’ implementation. Overall, these results provide a hypothesis-generating framework for exploration in larger samples of Australian clinicians to inform the development of implementation interventions in this area.

## Figures and Tables

**Table 1:** Kendall’s Tau-b correlations with the reported time dedicated to implementing ERP for youth.

Variables	Reported Time Dedicated to Implementing ERP for Youth (τ)	
	Within-Session	Between-Session
Demographics		
Age in Years	−0.19	−0.34 *
Female Gender ^a^	0.01	0.14
Capabilities		
Level of Education	0.39 *	0.10
Years of Experience as a Clinician	−0.34 *	−0.27
Experience Treating Youth with OCD	0.29	0.28
Training in Specialised CBT for OCD	0.39 *	0.37 *
Knowledge of ERP for Youth	0.48 **	0.25
Observed Adherence Delivering ERP for Youth	0.39 *	0.38 *
Observed Competence Delivering ERP for Youth	0.43 **	0.34 *
Opportunities		
Working in Private Practice ^b^	0.28	0.05
Working with Adolescents ^c^	−0.08	0.35 *
Recent Supervision in the Treatment of Youth with OCD	0.49 **	0.37 *
Motivations		
Profession as a Psychologist ^d^	0.50 **	0.35
Negative Beliefs about ERP for Youth	−0.49 ***	−0.34 *
Confidence Using ERP for Youth	0.55 ***	0.40 *
Time Intended to Dedicate to Implementing Within-Session ERP for Youth	0.47 **	0.44 **
Time Intended to Dedicate to Implementing Between-Session ERP for Youth	0.15	0.62 ***

Note. ^a^ 1 = male; 2 = female. ^b^ Dummy-coded as 1 = other; 2 = private practice. ^c^ 1 = children (i.e., <12 years old); 2 = adolescents (i.e., 12–18 years old). ^d^ Dummy-coded as 1 = social worker; 2 = psychologist. * *p* < 0.05. ** *p* < 0.01. *** *p* < 0.001.

**Table 2:** Mann–Whitney *U* tests comparing the typical (*n* = 25) and experienced subsamples (*n* = 13) across key variables.

Variables	Mean Rank			
	Typical Clinicians	Experienced Clinicians	*U*	*r*
Capabilities				
Knowledge of ERP for Youth	14.92	28.31	48.00 ***	−0.58
Observed Adherence Delivering ERP for Youth ^a^	9.75	17.88	31.50 **	−0.54
Observed Competence Delivering ERP for Youth	9.82	17.79	32.50 **	−0.52
Motivations				
Negative Beliefs about ERP for Youth	23.15	11.35	56.50 ***	−0.52
Confidence Using ERP for Youth	12.00	28.15	11.00 ***	−0.77
Time Intended to Dedicate to Implementing Within-Session ERP for Youth	7.92	18.50	82.00 *	−0.35
Time Intended to Dedicate to Implementing Between-Session ERP for Youth	8.63	16.38	105.50	−0.22
Implementation Behaviour				
Reported Time Dedicated to Implementing Within-Session ERP for Youth	15.23	22.69	12.00 ***	−0.72
Reported Time Dedicated to Implementing Between-Session ERP for Youth	16.30	20.88	25.50 **	−0.55

Note. Exact two-tailed significance, uncorrected for ties, was reported. ^a^ Both typical (*n* = 14) and experienced (*n* = 12) subsample sizes differed for role-play data. * *p* < 0.05. ** *p* < 0.01. *** *p* < 0.001.

## Data Availability

The data are available from the corresponding author upon request. The data are not currently publicly available as the broader research project is ongoing.

## Appendix A

**Table A1:** Relevant medians and interquartile ranges for typical and experienced subsamples.

Variables	*Mdn* (IQR)	
	Typical Clinicians	Experienced Clinicians
Observed Adherence Delivering ERP for Youth	11.00 (4.25)	13.50 (2.50)
Observed Competence Delivering ERP for Youth	2.10 (0.60)	2.77 (0.34)
Time Intended to Dedicate to Implementing Within-Session ERP for Youth	47.50 (61.00)	80.00 (35.00)
Time Intended to Dedicate to Implementing Between-Session ERP for Youth	55.00 (76.00)	90.00 (45.00)
Reported Time Dedicated to Implementing Within-Session ERP for Youth	10.00 (31.00)	77.50 (35.00)
Reported Time Dedicated to Implementing Between-Session ERP for Youth	10.00 (23.00)	80.00 (38.00)
